# Supplementary material for: Human microbiome-derived peptide affects the development of experimental autoimmune encephalomyelitis via molecular mimicry
Source: eBioMedicine. 2024 Dec 25;111:105516. doi: 10.1016/j.ebiom.2024.105516 (PMC11732510; doi:10.1016/j.ebiom.2024.105516)
Supplement: Supplementary Figs. S1–S6 [file mmc1.docx]

**Supplementary Materials for**

***Human microbiome-derived peptide affects the development of experimental autoimmune encephalomyelitis via molecular mimicry***

**Authors****:**

Xin Ma^1, #^, Jian Zhang^2, #^, Qianling Jiang^1^, Yong-Xin Li^2, *^ and Guan Yang^1,3 *^

**Affiliations:**

1. Department of Infectious Diseases and Public Health, Jockey Club College of Veterinary Medicine and Life Sciences, City University of Hong Kong, Kowloon, Hong Kong SAR, China.

2. Department of Chemistry and The Swire Institute of Marine Science, The University of Hong Kong, Pokfulam Road, Hong Kong SAR, China.

3. Shenzhen Research Institute, City University of Hong Kong, Shenzhen, China.

#equal contribution

*Corresponding Authors: Yong-Xin Li, Guan Yang.

**Email:** yxpli@hku.hk, gyang25@cityu.edu.hk


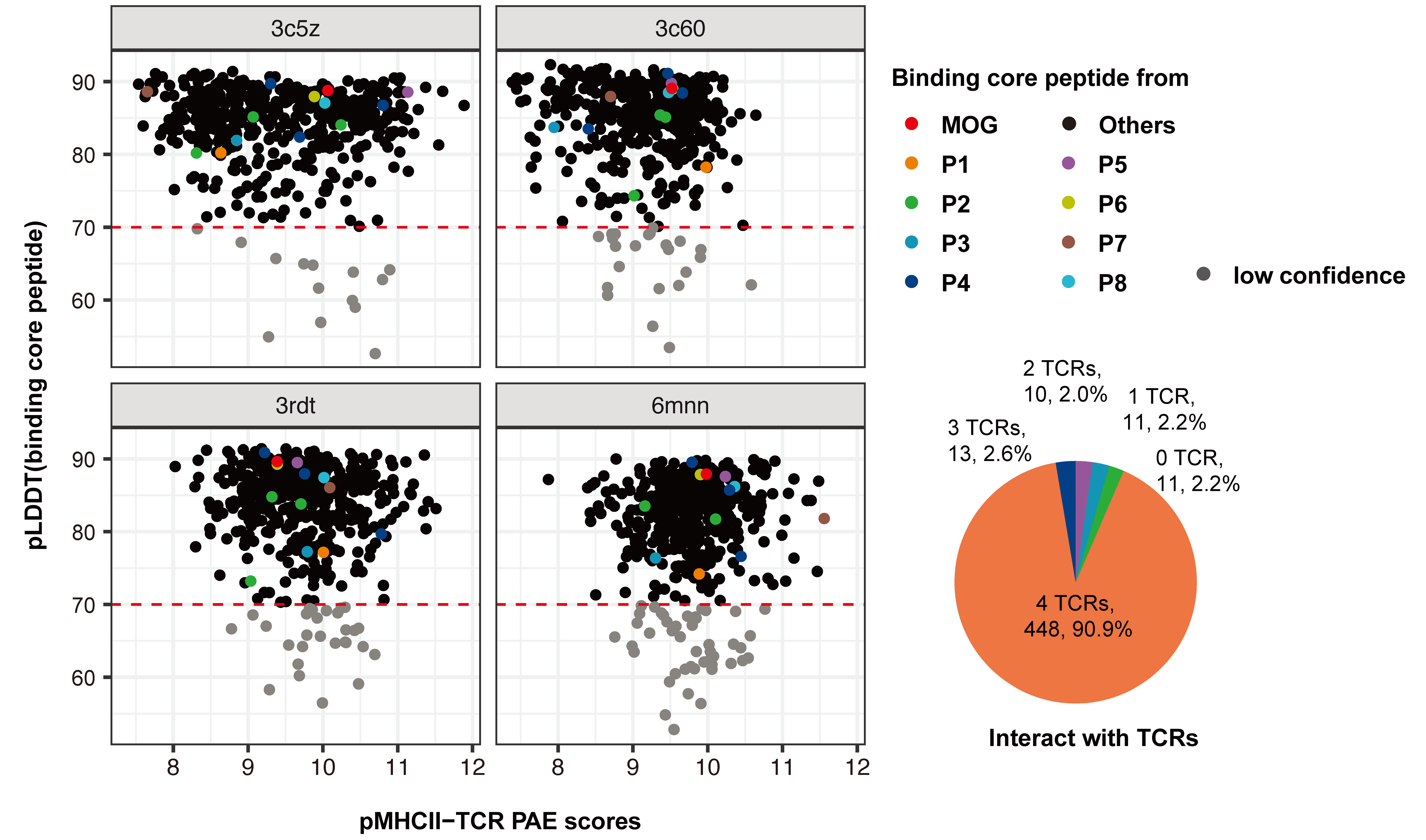


Supplementary figure 1. The confidence of microbiome-derived ligands’ binding core peptide-MHC II interact with 4 types of TCR predicted TCRDock. The scatter plots show the pLDDT value of a peptide versus an averaged inter-chain PAE of peptide-MHCII-TCR complexes. Two binding core peptides of MOG highlighted in green and orange, were shown as controls. Microbiome-derived peptides were colored in red, grey, and grey based on the modeling confidence. The complex with a binding core peptides’ pLDDT ≥ 70 and pMHCII-TCR PAE ≤ 8 was considered confident, and the complex with a binding core peptides’ pLDDT ≥ 70 and pMHCII-TCR PAE > 8 was considered as low confidence. The complex with a binding core peptides’ pLDDT < 70 was considered very low confidence. The pie chart shows a binding core peptide could interact with how many TCRs with confidence or low confidence: 91% of peptides could interact with 4 specific TCRs.





**Supplementary figure 2.** Per-residue confidence scores (pLDDT) of each chain for peptide-MHC-TCR complexes using templates with PDB id: 3c5z (A), 3c60 (B), 3rdt (C), 6mnn (D).


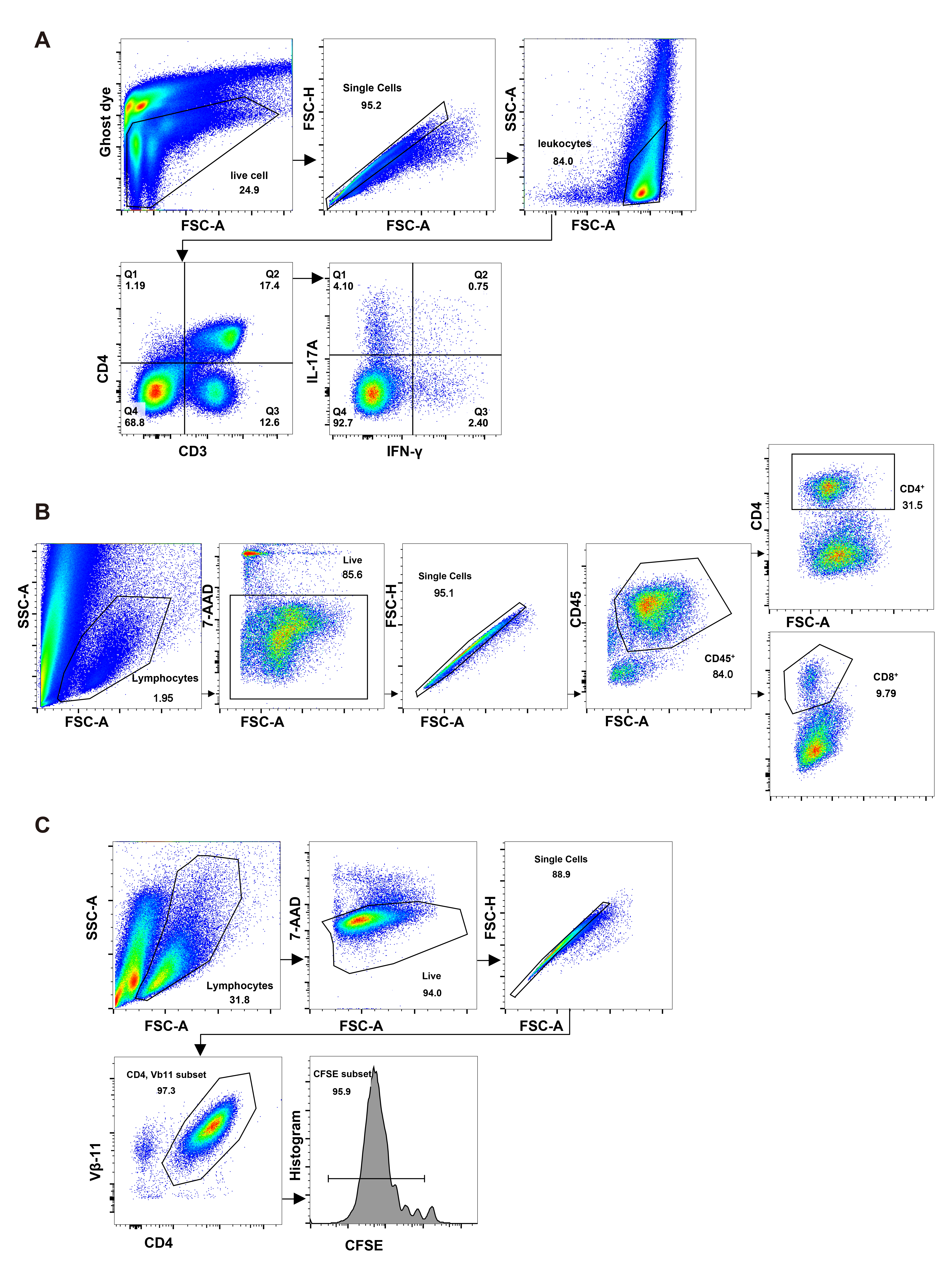
**Supplementary figure 3. Gating strategies.** (A) Gating strategy for cytokine response detection. (B) Gating strategy for the analysis of leukocytes in the spinal cord. (C) Gating strategy for the identification of CFSE population.


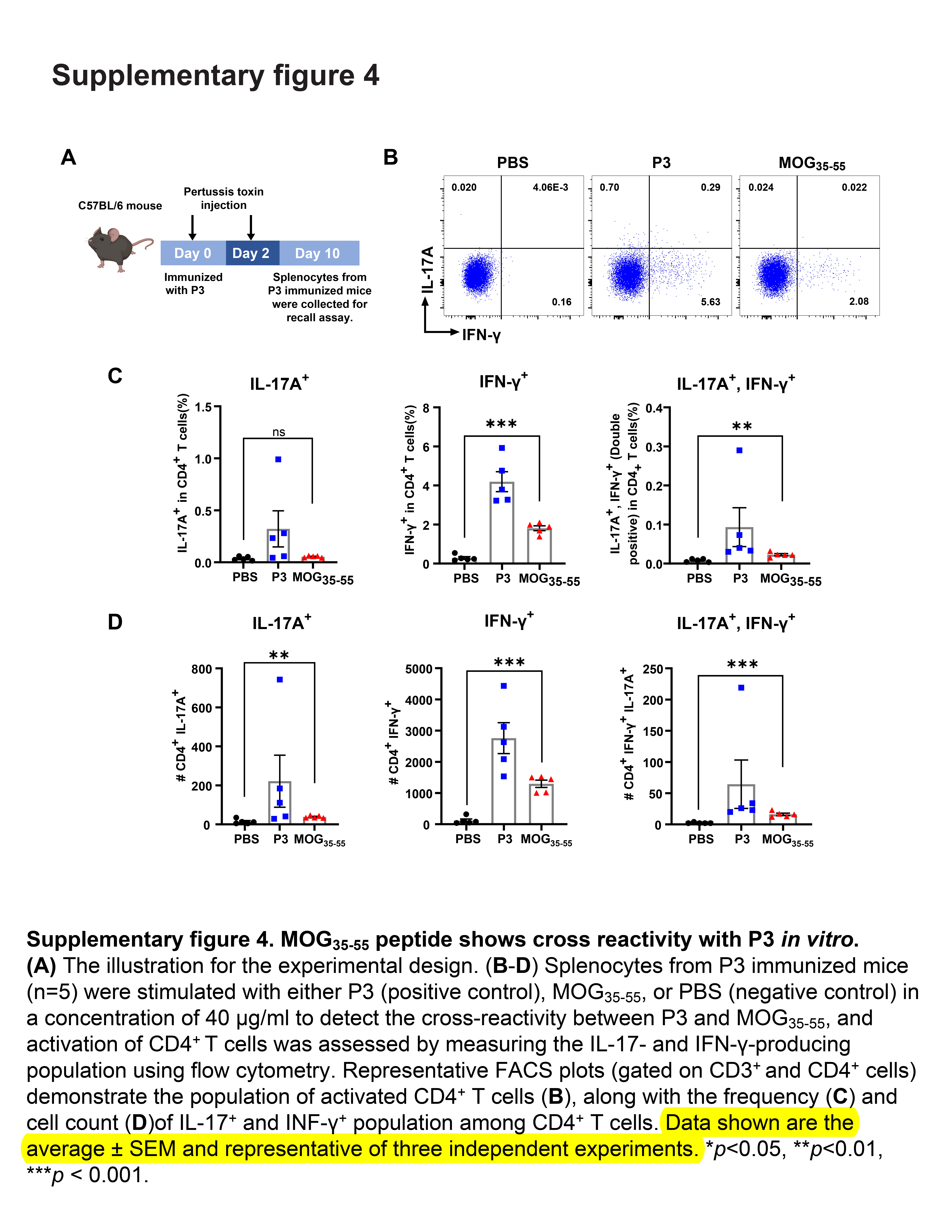
**Supplementary figure 4. MOG_35-55_ peptide shows cross reactivity with P3-specific T cells *in vitro*.** (A) Illustration of the experimental design. (B-D) Splenocytes from mice (n = 5) immunized with P3 were stimulated with either P3 (positive control), MOG_35-55_, or PBS (negative control), using a concentration of 20 μg/ml. The populations producing IL-17A and IFN-γ were assessed via flow cytometry. Representative FACS plots (B), the frequency (C), and cell count (D) of IL-17A^+^ and IFN-γ^+^ cells within the CD4^+^ T cell population are presented. Results show one experiment that is representative of three independent experiments. The data shown are the average ± SEM. ***p* < 0.01, ****p* < 0.001.



**Supplementary figure 5. P3 in combination with MOG_35-55_ increase T cells infiltration in CNS.** Spinal cord samples were collected at 17 dpi and T cell population within the CNS was assessed by flow cytometry. Representative FACS plots demonstrate the population of infiltrated CD4^+^ (A) and CD8^+^ (B) T cells. Data shown are the average ± SEM, n = 8 in each group. **p* < 0.05.



**Supplementary figure 6. P3 in combination with MOG_35-55_ exacerbates the development of EAE.** (A) Mean EAE clinical scores and (B) Kaplan-Meier curve of disease-free survival for each group of mice (n = 5) indicate that the combination of P3 and a low dose MOG_35-55_ enhances EAE progression during the early phase of the disease. Data are presented as the average ± SEM. **p* < 0.05.
